# Design of a multi-center immunophenotyping analysis of peripheral blood, sputum and bronchoalveolar lavage fluid in the Subpopulations and Intermediate Outcome Measures in COPD Study (SPIROMICS)

**DOI:** 10.1186/s12967-014-0374-z

**Published:** 2015-01-27

**Authors:** Christine M Freeman, Sean Crudgington, Valerie R Stolberg, Jeanette P Brown, Joanne Sonstein, Neil E Alexis, Claire M Doerschuk, Patricia V Basta, Elizabeth E Carretta, David J Couper, Annette T Hastie, Robert J Kaner, Wanda K O’Neal, Robert Paine III, Stephen I Rennard, Daichi Shimbo, Prescott G Woodruff, Michelle Zeidler, Jeffrey L Curtis

**Affiliations:** Research Service, VA Ann Arbor Healthcare System, Ann Arbor, MI 48105, USA; Pulmonary & Critical Care Medicine Section, Medicine Service, VA Ann Arbor Healthcare System, Ann Arbor, MI 48105 USA; Pulmonary & Critical Care Medicine Division, Department of Internal Medicine, University of Michigan Health System, Ann Arbor, MI 48109 USA; Center for Environmental Medicine, Asthma, and Lung Biology, Chapel Hill, NC 27599 USA; Center for Airways Disease, Department of Medicine, University of North Carolina at Chapel Hill, Chapel Hill, NC 27599 USA; Marsico Lung Institute/University of North Carolina Cystic Fibrosis Center, University of North Carolina at Chapel Hill, Chapel Hill, NC 27599 USA; Center for Genomics and Personalized Medicine, Wake Forest University, Winston-Salem, NC 27157 USA; Division of Pulmonary and Critical Care Medicine, Departments of Medicine and Genetic Medicine, Weill Cornell Medical College, New York, NY 10021 USA; Division of Pulmonary, Department of Internal Medicine, University of Utah Health Sciences Center, Salt Lake City, UT 84112 USA; Pulmonary, Critical Care, Sleep and Allergy Division, Department of Internal Medicine, University of Nebraska Medical Center, Omaha, NE 68198 USA; Department of Medicine, Columbia University Medical Center, New York, NY 10032 USA; Division of Pulmonary, Critical Care, Sleep and Allergy, Department of Medicine, University of California at San Francisco, San Francisco, CA 94143 USA; Division of Pulmonary, Critical Care, and Sleep Medicine, David Geffen School of Medicine, University of California at Los Angeles, Los Angeles, CA 90095 USA; Pulmonary and Critical Care Medicine Section (506/111G), Department of Veterans Affairs Healthsystem, 2215 Fuller Road, Ann Arbor, MI 48105-2303 USA

**Keywords:** Human, COPD, Flow cytometry, Sputum, Bronchoalveolar lavage, Immunophenotyping

## Abstract

**Background:**

Subpopulations and Intermediate Outcomes in COPD Study (SPIROMICS) is a multi-center longitudinal, observational study to identify novel phenotypes and biomarkers of chronic obstructive pulmonary disease (COPD). In a subset of 300 subjects enrolled at six clinical centers, we are performing flow cytometric analyses of leukocytes from induced sputum, bronchoalveolar lavage (BAL) and peripheral blood. To minimize several sources of variability, we use a “just-in-time” design that permits immediate staining without pre-fixation of samples, followed by centralized analysis on a single instrument.

**Methods:**

The Immunophenotyping Core prepares 12-color antibody panels, which are shipped to the six Clinical Centers shortly before study visits. Sputum induction occurs at least two weeks before a bronchoscopy visit, at which time peripheral blood and bronchoalveolar lavage are collected. Immunostaining is performed at each clinical site on the day that the samples are collected. Samples are fixed and express shipped to the Immunophenotyping Core for data acquisition on a single modified LSR II flow cytometer. Results are analyzed using FACS Diva and FloJo software and cross-checked by Core scientists who are blinded to subject data.

**Results:**

Thus far, a total of 152 sputum samples and 117 samples of blood and BAL have been returned to the Immunophenotyping Core. Initial quality checks indicate useable data from 126 sputum samples (83%), 106 blood samples (91%) and 91 BAL samples (78%). In all three sample types, we are able to identify and characterize the activation state or subset of multiple leukocyte cell populations (including CD4+ and CD8+ T cells, B cells, monocytes, macrophages, neutrophils and eosinophils), thereby demonstrating the validity of the antibody panel.

**Conclusions:**

Our study design, which relies on bi-directional communication between clinical centers and the Core according to a pre-specified protocol, appears to reduce several sources of variability often seen in flow cytometric studies involving multiple clinical sites. Because leukocytes contribute to lung pathology in COPD, these analyses will help achieve SPIROMICS aims of identifying subgroups of patients with specific COPD phenotypes. Future analyses will correlate cell-surface markers on a given cell type with smoking history, spirometry, airway measurements, and other parameters.

**Trial registration:**

This study was registered with ClinicalTrials.gov as NCT01969344.

## Background

Chronic obstructive pulmonary disease (COPD) is a chronic disease that is defined by the presence of airflow limitation that is not fully reversible. COPD is the third-leading cause of death in the United States [1] and is projected to become the fifth-leading cause of disease burden worldwide by the year 2020 [2]. COPD is associated with a persistent inflammatory immune response in the lungs in response to inhaled oxidants, including indoor air pollution from biomass fuels and cigarette smoke [3]. However, COPD is a complex disease involving more than just airflow obstruction. In many patients, COPD is associated with systemic manifestations or co-morbidities that can result in reduced quality of life and increased mortality [4]. There is significant heterogeneity between COPD patients with regard to symptoms, clinical characteristics and co-morbidities, physiology, imaging, response to therapy, decline in lung function and survival [5]. Identifying subtypes of patients may lead to more targeted and personalized therapeutic treatment.

The Subpopulations and Intermediate Outcomes in COPD Study (SPIROMICS) is an ongoing multicenter observational study funded by the National Heart, Lung and Blood Institute, NIH, with a primary goal of identifying homogenous subgroups of patients with COPD [6]. SPIROMICS is currently assembling a prospective cohort of 3200 participants for the collection and analysis of extensive phenotypic, biomarker, genetic, genomic and clinical data. In a subset of 300 subjects, peripheral blood, sputum and bronchoalveolar lavage (BAL) is being collected to immunophenotype multiple cell populations using flow cytometry. Cell populations of interest, including neutrophils, monocytes, macrophages, eosinophils, dendritic cells, T cells and B cells, as well as their activation states, are being identified using a 12-color antibody panel.

Immunofluorescence analysis by flow cytometry is the gold-standard for defining leukocyte populations. However, due to the complexity and sensitivity of flow cytometry, there are significant methodological hurdles when applied to a multicenter trial. Early studies from the Multicenter AIDS Cohort Study (MACS), in which four flow cytometry laboratories analyzed identical peripheral blood specimens, identified the importance of standardizing the model of flow cytometer, the antibody reagents and fluorochromes, the procedure for sample preparation and the procedure for sample analysis [7]. Many multicenter trials continue to stain the sample locally and use the flow cytometry instruments that are available at each participating institution [8-10]. Other studies have employed fixatives to stabilize receptor expression before staining, particularly in peripheral blood samples, with subsequent centralized core staining and flow cytometric analysis has also been explored; however preservation of individual surface markers by this approach was variable [11,12].

The goal of this sub-study is to provide state-of-the-art immunophenotyping of sputum, blood and BAL to be correlated with the abundance of other clinical, radiographic, physiological, genetic and biomarker data being collected on this cohort. We took the approach of “just-in-time” provision of reagents from a centralized Immunophenotyping Core, which prepares the 12-color antibody panels and ships them to the institutions as needed. On the day when samples are collected at each clinical site, they are stained without pre-fixation, then fixed and shipped overnight express on cold packs to the Immunophenotyping Core for data acquisition and analysis on a single flow cytometer. The choice of leukocyte cell types and their receptors was based on a series of pre-specified hypotheses plus research interests of the coauthors. This interim report demonstrates the feasibility of our approach, which may be of value in the design of multicenter trials in COPD or other disease states.

## Materials and methods

### Ethics statement

All clinical investigations are conducted according to the principles of the Declaration of Helsinki. The study protocol was approved by the individual institutional review boards (Columbia University; Weill Cornell Medical College; University of California Los Angeles; University of California San Francisco; University of Michigan, University of Utah; Wake Forest University). All participants understand the purpose of the study and provide written informed consent before they undergo any research activities or procedures.

### Study design and logistics

A subgroup of 50 subjects from each of six clinical sites (total *n* = 300) is being enrolled from the parent SPIROMICS study. The enrollment strata for the bronchoscopy sub-study are described in Table 1. Subjects participate in this sub-study during two separate visits. At the first visit, a sputum sample is collected by induction. In the second visit, which takes place two to four weeks later, peripheral blood and bronchoalveolar lavage samples are collected.

**Table 1:** **Planned subject enrollment distribution by strata**

	**Never-smokers**	**Smokers without airflow obstruction**	**Smokers with mild to moderate COPD**	**Smokers with severe COPD**
**Smoking status**	<1 pack year	>20 pack years	>20 pack years	>20 pack years
**Lung function**	FEV_1_/FVC >0.7	FEV_1_/FVC >0.7	FEV_1_ > 50% predicted	50% > FEV_1_ > 30% predicted
**Sample size**	N = 60 (20%)	N = 60 (20%)	N = 140 (47%)	N = 40 (13%)

The SPIROMICS clinical sites are broadly distributed geographically, being located in Ann Arbor (University of Michigan); Los Angeles (University of California); New York City (Columbia & Cornell Universities); Salt Lake City (University of Utah); San Francisco (University of California); and Winston-Salem (Wake Forest University). To assure efficient communication, we follow a standardized notification process. Study coordinators at the Clinical Centers are required to notify both the Immunophenotyping Core and their local collaborating laboratory as soon as the first bronchoscopy sub-study visit is scheduled, so that antibody panels can be prepared and shipped overnight to that site. Notification occurs by email to multiple individuals at both the clinical sites and the Immunophenotyping Core, to minimize the chance that an absence of one individual will interfere with the tight shipping schedule. The individuals primarily responsible for this protocol at both the Clinical Centers and the Immunophenotyping Core follow a strict policy of immediately “replying to all” at both sites, confirming receipt of each email and repeating back the received information, to affirm that the message has been received correctly.

Next, assay tubes, each containing all the antibodies (or isotype controls) for a given cell type or groups of related cell types, are prepared by the Immunophenotyping Core. Each tube is identified using labels supplied by the SPIROMICS Genomics and Informatics Core (GIC) at the University of North Carolina. These labels are specific to sample type and subject, but do not include the SPIROMICS-wide subject identifier. Thus, the Immunophenotyping Core is blinded to any clinical information about the subjects at the time of the flow cytometry analysis, as the only link between sample labels and subject IDs is held by the GIC.

Once antibodies are aliquoted, tubes are capped and centrifuged at 300 × *g* for 5 minutes, the tubes are placed in wire tacks, which are wrapped in aluminum foil to shield them from light, and are stored at 4°C until shipment. Tubes are affixed with a sample-specific label (which can later be matched by the GIC to specific subject information) and then are shipped from the Immunophenotyping Core to the Clinical Centers between 3–7 days before the scheduled appointment. The Immunophenotyping Core notifies the Clinical Center by email that the assay tubes have been shipped and provides the tracking information. When assays are shipped, the Immunophenotyping Core records the assay ID number from the labels, plus the date and Clinical Center to which that particular assay was shipped. This information is transmitted to the GIC.

An identical process of email communication between the Clinical Centers, the Immunophenotyping Core and the GIC is followed once the sample has been collected, stained and fixed. Thus, the GIC records the date on which an assay was shipped; the Immunophenotyping Core records the date on which it was received, facilitating prompt location of any assays that become delayed or lost in transit. To reduce the chance that assays will not be properly chilled during transit, shipping in either direction is permitted only Monday through Thursdays. Additionally, care is taken to assure that the timing of holidays (especially Federal, given that the Immunophenotyping Core is a VA facility) is considered before shipments are released.

### Biospecimen collection

Sputum induction was performed according to the methods of Alexis et al. [13]. Personnel at the clinical sites involved in sputum induction and sample processing received onsite, in-person training from Dr. Alexis. Briefly, subjects undergo seven-minute exposures to increasing concentrations of aerosolized hypertonic saline by inhaling via a mouthpiece. To minimize oral contamination of induced sputum specimens, subjects are asked to rinse their mouths with water, to blow their noses and to clear their throat at the end of each inhalation period, then to “cough from their chest” and immediately expectorate into a cup without holding the specimen in their mouths. To assure subject safety, spirometry is performed during the inhalation period and again at the end of each seven minute exposure. The saline concentrations used and frequency of spirometric testing vary according to the subject’s baseline forced expiratory volume in 1 second (FEV1). Subjects with baseline FEV1 ≥ 50% predicted inhale 3%, 4% and 5% saline, and undergo spirometry two minutes into each exposure and at the end of the exposure. By contrast, subjects with baseline FEV_1_ < 50% predicted inhale 0.9% and 3% saline, and undergo spirometry at 1, 2, 5 and 7 minutes of exposure. If at any point the FEV1 decreases by >20% from baseline, the induction is stopped; otherwise, subjects either continue the current exposure period or proceed to the next saline concentration. Sputum samples were kept on ice throughout the induction procedure and processed for immunophenotyping immediately following collection.

At the second visit, during which blood and BAL are collected, post-bronchodilator FEV_1_ is measured before any procedures. Only subjects with an FEV1 > 30% predicted that day are allowed to participate in the bronchoscopy visit. At the time of IV placement, blood is drawn into a 10 ml heparin plasma tube, and immediately to the laboratory for immunophenotyping staining. A complete blood count (CBC) is also collected, and processed by the medical center clinical laboratory.

The BAL sample for Immunophenotyping is only one portion of collection of multiple samples that comprise the entire Bronchoscopy sub-study. BAL is performed in the right middle lobe and lingula by instilling two aliquots of 40 mL and one aliquot of 50 mL of sterile saline per lobe (i.e., 130 mL per lobe, total volume = 260 mL per subject), which is withdrawn by gentle manual suction. The BAL return is collected into specifically designated specimen traps, kept on ice. The BAL from both lung sites was pooled and used for immunophenotyping.

### Antibody panels

We designed 12-color monoclonal antibody panels with isotype controls to analyze multiple leukocyte populations. The antibody panel, with clones listed in parentheses, is shown in Table 2. The panels for BAL and sputum differ from the panel for peripheral blood in that they do not contain antibodies for basophils or endothelial cells. Additionally, the sputum panel does not contain antibodies for dendritic cells or B cells. These choices were made based on pilot data from our laboratory indicating that these cell types were present in such low frequency as to be impractical to identify. Antibodies and isotype-matched controls were directly conjugated to either fluorescein isothiocyanate (FITC), eFluor 450, phycoerythrin (PE), phycoerythrin-cyanine 5 (PE-Cy5), peridinin chlorophyll protein-cyanin 5.5 (PerCP-Cy5.5), phycoerythrin Texas red (PE-TR), phycoerythrin-cyanine 7 (PE-Cy7), allophycocyanin (APC), allophycocyanin-cyanine 7 (APC-Cy7), BD Horizon™ V500 (V500), Pacific Blue, Alexa Fluor 700 (AF 700), and QDot® 655. Vendors from which antibodies were purchased include Biolegend (San Diego, CA), eBioscience (San Diego, CA), BD Biosciences (San Jose, CA), R&D Systems (Minneapolis, MN), Miltenyi Biotec (Auburn CA), and Invitrogen (Carlsbad, CA). Antibodies against CX3CR1, CD133, and their respective isotypes, were purchased unconjugated, and we used Lightning-Link antibody labeling kit (Novus Biologicals, Littleton, CO) to conjugate these antibodies to APC-Cy7 and Atto 700, respectively.

**Table 2:** **Standardized antibody panel**

**Leukocyte population**												
	**Fluorochrome (directly conjugated to monoclonal antibody)**											
	**FITC**	**PE**	**PE-Cy5**	**PerCP-Cy5.5**	**PE-TR**	**PE-Cy7**	**APC**	**APC-Cy7**	**V500**	**Pacific blue**	**AF 700**	**QDot 655**
	**Surface antigen detected with monoclonal antibody clone used (italicized)**											
Dendritic cells^#^	BDCA-2 *AC144*	BDCA-1 *AD5-8E7*	CD123 *6H6*	CCR2 *TG5*	CD45 *HI30*	CD103 *B-Ly*7	BDCA-3 *AD5-14H12*	CX3CR1 *2A9-1*	HLA-DR *L243*	CD11c *3.9*	CD11b *CBRM1/5*	CD3 *7D6* & CD19 *SJ25-C1*
Mø & monocytes	CD14 *HCD14*	CCR6 *R6H1*	CD16 *3G8*	CCR2 *TG5*	CD45 *HI30*	TLR2 *T2.5*	CD206 *15-2*	CX3CR1 *2A9-1*	HLA-DR *L243*	CD11c *3.9*	CD11b *CBRM1/5*	
Mø & monocytes	CD14 *HCD14*	Axl *108737*	CD16 *3G8*	DC-Sign *9E9A8*	CD45 *HI30*	TLR4 *HTA125*	Mertk *125518*		HLA-DR *L243*	CD11c *3.9*	CD11b *CBRM1/5*	
Basophils^*#^	CD33 *HIM3-4*	CD9*C3-3A2*	CD13 *TuK1*	CD203c *NP4D6*	CD45 *HI30*	CD63 *H5C6*	CD22 *HIB22*	CD34 *581*	HLA-DR *L243*	CD69 *FN50*	CD11b *CBRM1/5*	CD19 *SJ25-C1*
Eosinophils	CD49d *9 F10*	CDw125 *A14*	CD16 *3G8*	CD69 *FN50*	CD45 *HI30*		CCR3 *5E8*	CD34 *581*			CD11b *CBRM1/5*	
Neutrophils	CD177 *MEM-166*	CD16b *CLB-gran 11.5*	CXCR1 *8 F1*	CD66b *G10F5*	CD45 *HI30*	TLR4 *HTA125*	CXCR2 *5E8*	CD10 *HI10a*		TLR2 *T2.5*	CD11b *CBRM1/5*	
Endothelial cells^*#^	CD33 *HIM3-4*	CD146 *SHM-57*	VCAM-1 *STA*	VEGFR2 *HKDR-1*	CD45 *HI30*	PECAM *WM59*	CD144 *16B1*	CD34 *581*		ICAM-1 *HCD54*	CD133 *293C3*	CD3*7D6* & CD19 *SJ25-C1*
B cells^#^	IgD *IA6-2*	CD80 *2D10.4*	CD86 *B7-2*	CD27 *O323*	CD45 *HI30*	CXCR4 *12G5*	CD23 *EBVCS-5*			CD20 *2H7*	CD38 *HIT2*	CD19 *SJ25-C1*
T cells	γδ -TCR *B1.1*	CTLA-4 *14D3*	CD28 *CD28.2*	CD27 *O323*	CD45 *HI30*	ICOS *ISA-3*	CD56 *CMSSB*	CD62L *DREG-56*	CD3 *UCHT1*	CD8 *OKT-8*	CD4 *OKT-4*	
T cells	CCR7 *150503*	CCR5 *T21/8*	CXCR3 *1C6*	CD27 *O323*	CD45 *HI30*		CD56 *CMSSB*	CD62L *DREG-56*	CD3 *UCHT1*	CD8 OKT-8	CD4 *OKT-4*	

*not included in BAL samples; ^#^not included in sputum samples.

Antibodies are centrally prepared for each clinical site at the Immunophenotyping Core at the VA Ann Arbor Healthcare System. Antibodies are aliquoted into flow tubes (BD #352008 and #35203; Becton Dickinson) which are capped, placed in a rack (Fisher #14-793-14), and covered with aluminum foil. Antibodies are shipped overnight in a Styrofoam box with multiple cold packs, frozen to −20°C, and typically arrive at the clinical site 1–3 days before the study visit.

### Staining of samples

At each clinical site, mucus plugs from the sputum sample are selected, weighed, and then incubated with 1× Sputolysin® Reagent (EMD Millipore, Billerica, MA) in a 37°C water bath for 20 minutes. Samples are washed and filtered before resuspending the cell pellet in Staining Buffer with FBS (BD #340345; BD Biosciences). The sputum assay antibody tubes from the Immunophenotyping Core are briefly centrifuged and then the entire sputum sample is divided among seven antibody tubes (100 μL per tube). Tubes are covered with aluminum foil and incubated at room temperature for 25 minutes with continuous shaking or rocking (depending on the equipment available at the clinical site laboratory). After the incubation, samples are washed with 2 mL Staining Buffer, centrifuged, and resuspended for storage in 2% freshly-prepared formaldehyde in PBS. Tubes are then stored at 4°C in a rack wrapped in aluminum foil before being shipped back to the Immunophenotyping Core.

BAL samples are centrifuged and resuspended in Staining Buffer, then 100 μL of the BAL sample is added to each of the BAL assay antibody tubes. Staining then proceeds as described above for sputum samples.

Blood tubes are inverted eight times and then 100 μL of the undiluted blood sample is added to each of the blood assay antibody tubes. Similar to the staining procedure for sputum and BAL, samples are incubated with the antibodies for 25 minutes. Next, to remove red blood cells, 2 mL of 1× BD Pharm Lyse (BD Biosciences) are added to each tube and incubated at room temperature for another 25 minutes. Samples are centrifuged and washed with Staining Buffer before being stored in 2% formaldehyde in a refrigerator wrapped in aluminum foil.

### Flow cytometry instrument setup and data acquisition

Samples are wrapped in aluminum foil and are shipped overnight in a Styrofoam box with cold packs to the Immunophenotyping Core. Upon arrival, samples are physically inspected and any issues (e.g. cracked tubes, inconsistent volumes, missing tubes) are recorded along with the sample ID. Samples are transferred to a 96-well U-bottom plate and data are acquired on an LSR II flow cytometer (BD Bioscience, San Jose, CA) with a High Throughput Sampler, equipped with the following four lasers, listed with their associated fluorochromes and filter sets: 488 nm blue laser (APC-Cy7: 735 nm long-pass (LP), 780/60 nm short band-pass (SBP); AF700: 690 nm LP, 730/45 nm SBP; APC: 660/20 nm SBP); 405 nm violet laser (Qdot655: 630 nm LP, 660/20 nm SBP; Horizon V500: 505 LP, 530/30 SBP; Pacific Blue: 450/50 SBP); 633 nm red HeNe laser (PerCP-Cy5.5: 685 nm LP, 695/40 SBP; FITC: 505 LP, 530/30 SBP); and a 561 nm yellow-green laser (PE-Cy7: 735 nm LP, 780/60 nm SBP; PE-Cy5: 635 nm LP, 670/30 nm SBP; PE-TR: 600 nm LP, 610/20 nm SBP; PE: 582/15 nm SBP).

Data are collected using FACSDiva software (BD Biosciences) with automatic compensation. CS&T Research beads (BD Biosciences) are used during instrument setup to maintain baseline performance values, thereby ensuring that the cytometer performed the same every time. Because cell yields varied, we collect all possible events to maximize our ability to detect rare populations.

Within 1–2 days after the sample has been run on the flow cytometer, we perform an initial quality check. We record the absolute number of CD45+ leukocytes in the sample and also look at the percentage of low side scatter cells, indicative of lymphocytes, and high side scatter cells, such as macrophages, neutrophils, and monocytes.

### Flow cytometry data analysis

Data are analyzed by two trained individuals (SC, VRS) using FlowJo software v.9.6.2 (Tree Star, Ashland, OR) on Macintosh Quad-Core Intel Xeon computers running OS X 10.10.1 (Apple; Cupertino, CA). We use pre-printed sample report forms and a flow analysis worksheet to standardize the process. All analyses undergo a secondary evaluation by a third individual (CMF) to help maintain consistency in the gating and analysis between samples. Data, including analysis files, are immediately backed up to DVD-R disks.

### Statistical analysis

Analyses were performed using GraphPad Prism 6.0 (GraphPad Software, Inc., La Jolla, CA) on a Macintosh Quad-Core Intel Xeon computer running OS 10.10 (Apple; Cupertino, CA). A two-tailed *p* value of < 0.05 was considered to indicate significance.

## Results

### Sample acquisition success rate

As of September 17, 2014, a total of 152 sputum samples and 117 blood/BAL samples had been returned to the Immunophenotyping Core. Disparity between collection of sputum samples and of blood plus BAL samples from what was planned appears to have resulted primarily from missed or yet to be completed return visits, and not from any adverse events related to the study procedures. For each specimen, we performed an initial analysis of the number of CD45+ cells in sputum, blood, and BAL, stratified by clinical site. Samples averaged 7.7×10^4^ ± 8.2×10^4^ (mean ± SD), 8.9×10^4^ ± 10.7×10^4^, and 20.3×10^4^ ± 36.6x10^4^ CD45+ events (leukocytes) per tube for sputum, BAL and blood, respectively. Samples containing fewer than 1×10^4^ leukocytes are deemed unusable and are not analyzed. Low cell yield was most typically seen in the sputum and BAL samples. We have deliberately not yet broken the codes linking samples to subjects, so that initial analyses are not biased by knowledge of clinical data.

Our preliminary analyses indicate that a total of 126 of 152 sputum samples (83%), 106 of 117 blood samples (91%) and 91 of 117 BAL samples (78%) provided usable data (Table 3). It seems likely that there may have been a learning curve in obtaining or processing the samples, particularly sputum. The percentage of usable sputum data has increased from 69% in 2012, to 88% in 2013, and 92% in 2014. BAL samples also showed a modest increase: 71% usable samples in 2012, 78% in 2013 and 80% in 2014 (data not shown).

**Table 3:** **Number and percentage of usable specimens by clinical site**

	**Site 1**	**Site 2**	**Site 3**	**Site 4**	**Site 5**	**Site 6**	**Total**
Sputum visits, *n*:	16	26	38	30	23	19	152
Usable specimens, *n*	13	21	34	27	17	14	126
Usable specimens,%	81%	81%	89%	90%	74%	74%	83%
Blood & BAL visits, *n*:	7	26	28	19	19	18	117
Usable blood specimens, *n*	6	22	25	19	17	17	106
Usable blood specimens, %	86%	85%	89%	100%	89%	94%	91%
Usable BAL specimens, *n*	6	21	22	12	15	15	91
Usable BAL specimens, %	86%	81%	76%	63%	79%	83%	78%

Processing errors may also account for some of the unusable data. The most commonly detected processing error was resuspension of the samples in an incorrect fixative volume, such that the concentration of paraformaldehyde, which must be held constant to stabilize the light scatter and antibody labeling, was likely much lower than the 2% specified in the protocol. Other less common errors result from shipping delays, which resulted in samples no longer protected by the ice packs; cracked tubes with low or missing sample; and improperly capped tubes, resulting in loss of entire samples. Although there was variation between clinical sites in the percentage of usable samples of different types, no site was routinely underperforming compared to the other sites (Table 3), supporting our impression that random errors, rather that systematic problems, were responsible for lost data.

### Duration between fixation and data acquisition has only minor effect on fluorescence intensity

**Figure 1:**
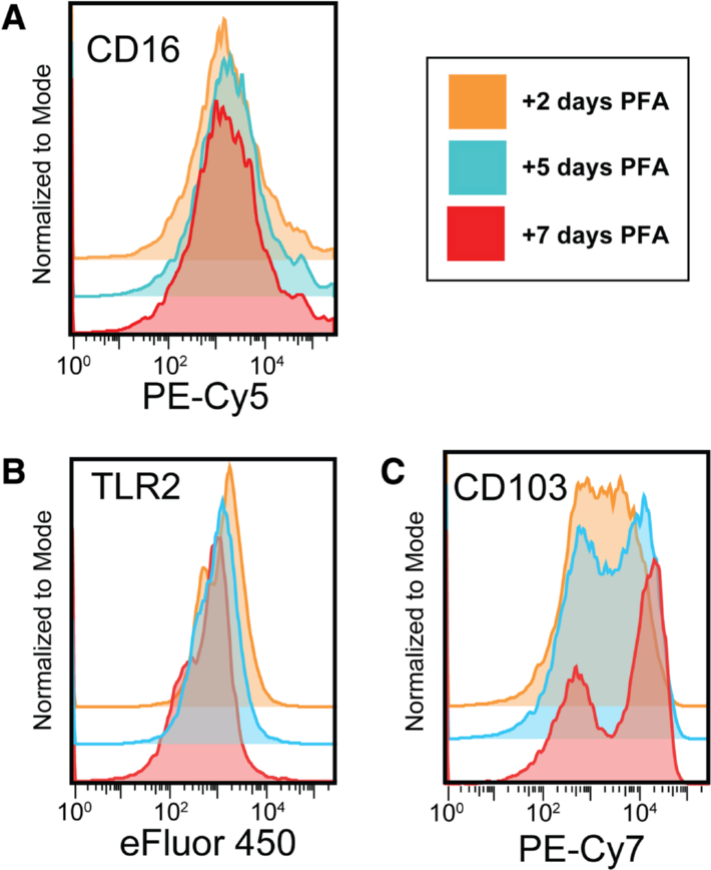
**Storage in PFA affects fluorescent intensity of specific surface receptor-monoclonal antibody combinations.** After addition of PFA, BAL samples were divided into three samples and data was acquired after storage at 4°C for either 2, 5, or 7 days. **A)** CD16 (and the majority of the surface antigens) showed no change in fluorescent intensity between days 2, 5, and 7. **B)** TLR2 showed an increase in fluorescent intensity at day 5 and at day 7. **C)** CD103 was the only receptor to show a decrease in fluorescent intensity at days 5 and 7.

**Figure 2:**
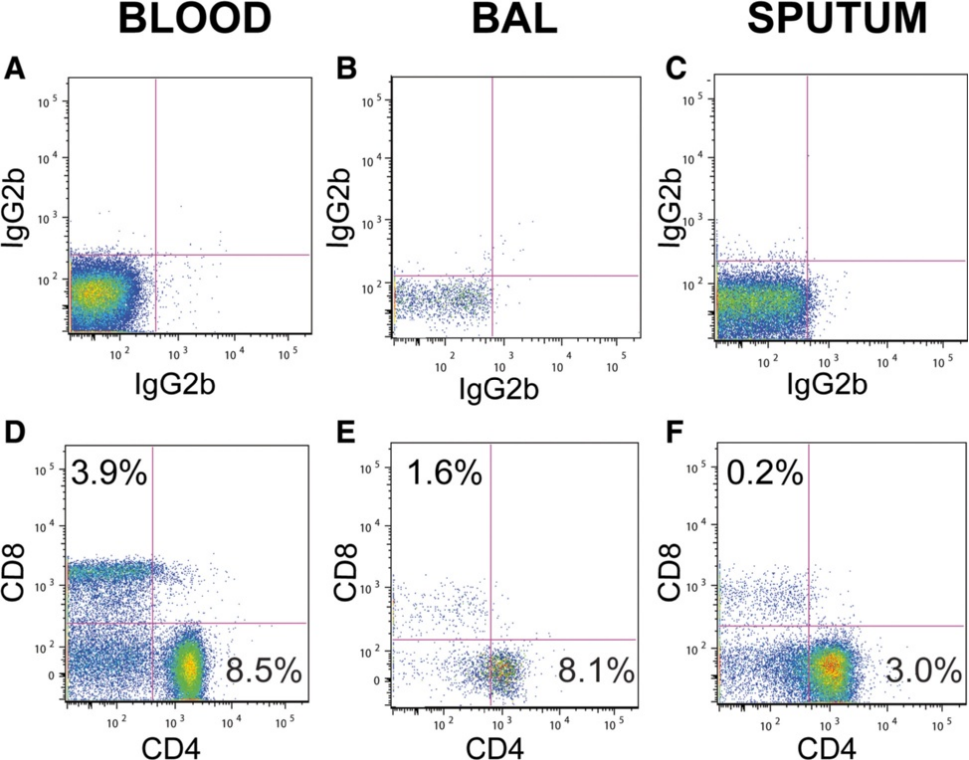
**Representative staining of CD4+ and CD8+ T lymphocytes in blood, BAL, and sputum.** Samples of peripheral blood **(A, D)**, BAL **(B, E)** and sputum **(C, F)** were gated on cells that were CD45+, CD3+, and had a low side-scatter. Next, using the isotype control **(A-C)**, quadrants denoting specific staining for CD4 (horizontal axis) and CD8 (vertical axis) were determined **(D-F)**. Numbers in the CD4+ and CD8+ quadrants represent the percent of each subset among all CD45+ cells.

One variable in this study is the duration between fixative addition and data acquisition on the flow cytometer. This duration typically varies between two and seven days, because sites that have study visits on Thursday or Friday are unable to ship specimens until the following Monday. To determine whether time of storage in PFA affected fluorescent intensity, in locally-performed pilot experiments, we analyzed blood and BAL samples at days 2, 5, and 7 post-PFA. Results indicated no difference in forward scatter, side scatter or CD45+ staining (not shown). Among 68 surface molecules, fluorescence intensity for 61 was either unaffected by storage in PFA, as shown for CD16 (Figure 1A), or had modest increases in specific staining and in the corresponding isotype control, such that there was no difference in overall positive staining. In blood specimens, antibodies against TLR4 (PE-Cy7), gamma-delta T cell receptor (FITC), and CCR7 (FITC) had increased specific fluorescence (relative to isotype control) from day 2 to day 5, but no further increase at day 7 (data not shown). In BAL specimens, antibodies against BDCA-1 (PE), CX3CR1 (APC-Cy7), and TLR2 (eFluor 450) also showed modest increases in specific fluorescence at days 5 and 7 (Figure 1B). Only CD103 (PE-Cy7) in the BAL samples exhibited decreased fluorescent intensity, which was seen at both days 5 and 7 (Figure 2C). Although small in absolute terms, these changes will need to be accounted for in future analyses. Importantly, however, staining for the vast majority of antigens were unaffected by duration of storage in PFA.

### Identification of T cell populations in blood, sputum and BAL

Lung T cells have been linked to progression of COPD, especially of emphysema, in multiple studies [14-19]. We used a combination of anti-CD45 (leukocyte common antigen), forward and side light scatter properties, and differential expression of lineage-specific surface markers to identify leukocyte populations, as has been previously shown to be effective for sputum leukocytes [13,20]. To identify CD4+ and CD8+ T cells, we first gated on CD45+ CD3+ cells with a low side scatter. Representative staining demonstrates the ability of this strategy plus comparison to isotype controls to distinguish CD4+ and CD8+ T cells in blood, BAL and sputum (Figure 2). The numbers in each quadrant indicate the percentage of that subset among all CD45+ cells. These numbers are consistent with what other studies have found in sputum [21], blood and BAL [22].

### Identification of macrophage populations in sputum and BAL

**Figure 3:**
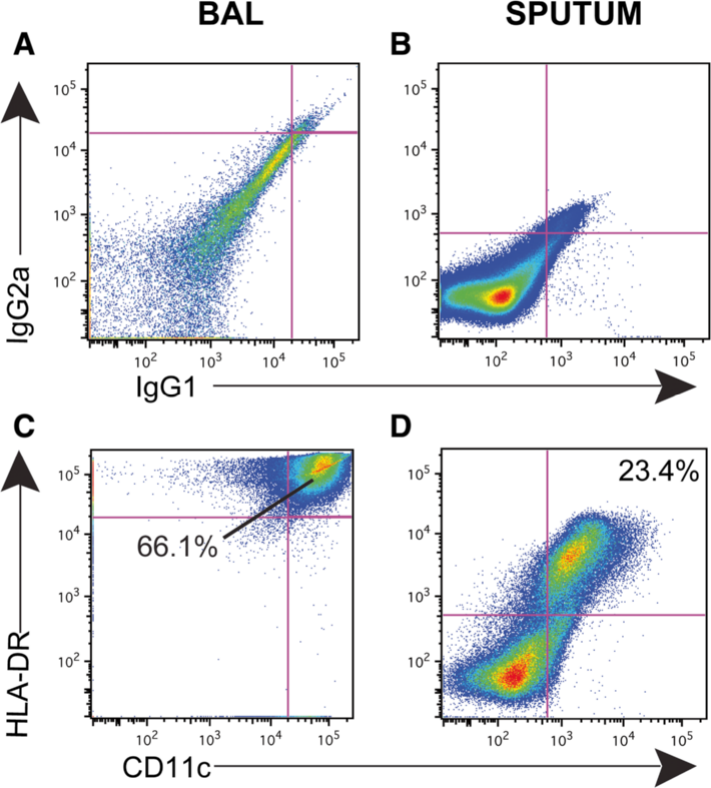
**Representative staining of macrophages in BAL and sputum.** To identify macrophages in samples of BAL **(A, C)** and sputum **(B, D)**, we gated on CD45+ auto-fluorescent cells and used isotype controls **(A,B)** to gate on cells specifically staining for HLA-DR+ CD11c + **(C, D)**. Numbers represent the percent of AMø among all CD45+ cells.

Alveolar macrophage (AMø) numbers are increased and their function is altered in COPD [23]. There is considerable interest in whether polarization of their gene products drives inflammation in COPD [24,25]. To identify AMø, we gated on CD45+, auto-fluorescent cells and then selected HLA-DR+ CD11c + cells (Figure 3). This approach was chosen after a preliminary analysis of a variety of alternative gating approaches, including use of Mertk (a putative pan-Mø marker in mice) [26], as the one giving the most unambiguous distinction of mature AMø. Although CD11b is included in the panel for analysis, we did not use it to gate on macrophages, as AMø have been shown to be negative for CD11b, unless activated, therefore their expression is not uniformly positive [27]. As expected, BAL samples contained the largest percentage of macrophages. As the numbers of subjects increase, this dataset will be analyzed further for correlation of macrophage surface receptor expression with spirometrically-defined disease severity and other clinical data.

### Identification of monocyte populations in blood, sputum and BAL

Monocytes constitute 5 to 10% of peripheral blood leukocytes where they circulate for several days before migrating into tissues. The degree to which recruitment of blood monocytes contributes to the expansion of the mononuclear phagocyte population in COPD is contested. Monocytes were originally divided into “classical” CD14++ CD16- cells and “nonclassical” CD14+ CD16+ cells. The classical monocytes were considered to be better at secreting proinflammatory cytokines and constitute the majority of all monocytes in healthy persons, whereas the nonclassical monocytes more closely resemble resident tissue macrophages [28-30]. A third subset of peripheral blood monocytes, termed “intermediate” monocytes are CD14++ CD16+ [31]. It is not clear whether these intermediate monocytes have a biologically meaningful role or are an intermediate step in the differentiation of monocytes but they have been shown to be increased in certain conditions including rheumatoid arthritis and severe asthma [32].

**Figure 4:**
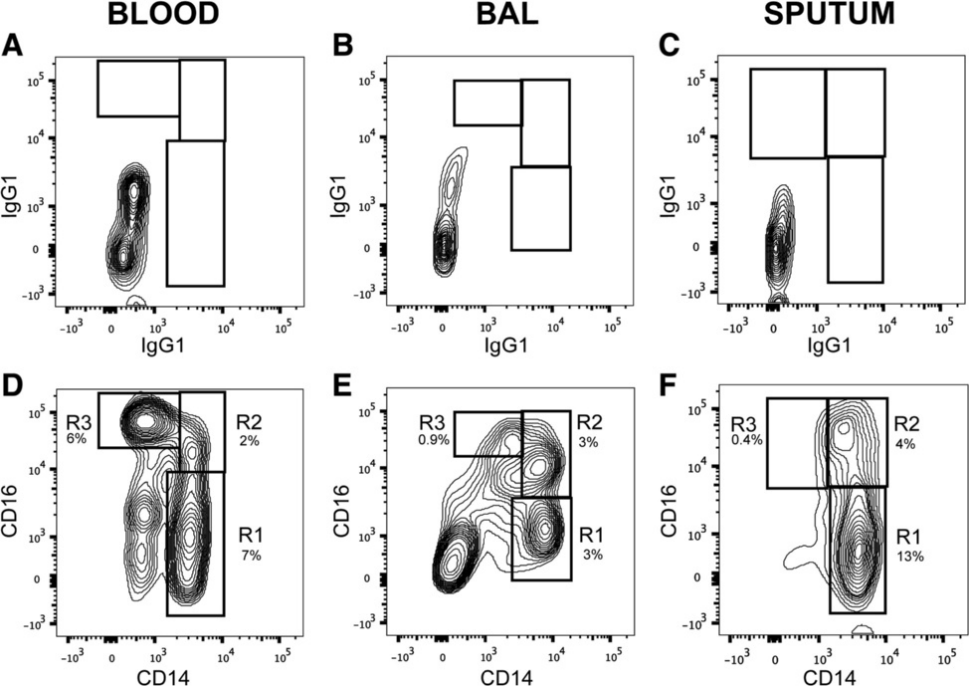
**CD14 and CD16 identify populations of monocytes in blood, BAL, and sputum.** To identify monocyte in samples of peripheral blood **(A, D)**, BAL **(B, E)** and sputum **(C, F)**, we first gated on non-autofluorescent, CD45+ cells with a medium side-scatter, then using isotype controls **(A-C)**, chose HLA-DR+ cells **(D-E)**. In all analyses, we then separated the monocytes into “classical” CD14++ CD16- cells (R1), “intermediate” CD14++ CD16+ (R2), and “nonclassical” CD14+ CD16+ (R3) cells. Numbers represent the percent of each monocyte subset among all CD45+ cells.

In our study, monocytes were readily identified in all three compartments using CD14 and CD16 antibodies, after gating on CD45+, non-autofluorescent cells with a medium side-scatter that were HLA-DR+, relative to isotype controls (Figure 4A-4C). We chose to define monocytes by HLA-DR+ staining, rather than expression of CD11b (typically positive on monocytes in peripheral blood) or CD11c (negative on blood monocytes, but potentially upregulated in GM-CSF-rich environments such as the lungs), so that we could independently analyze expression of the latter two markers in samples other than blood. As shown in the representative staining (Figure 4D), we can easily see distinct populations for the classical, non-classical, and intermediate monocytes in peripheral blood.

In the BAL and sputum, monocytes were distinct from alveolar macrophages due to their reduced size and granularity, evident by forward scatter and side scatter (data not shown). Monocytes were again divided into the three populations: classical, intermediate, and non-classical (Figure 4E, 4F). The populations appear to be more limited to the classical and intermediate phenotypes, reminiscent of a gating strategy developed by Brittan et al. [33] which uses the terms “inducible” and “resident”, respectively, due to the observation that LPS inhalation resulted in an increase in the CD14++ CD16- population, in comparison to a saline-treated group, but the CD14++ CD16+ population was unchanged between groups [33]. We also found that the monocytes from BAL and sputum were predominantly CD14++ CD16+, the so-called “resident” monocytes (Figures 4E, 4F).

### Identification of myeloid dendritic cell (mDC) populations in blood, sputum and BAL

In the present study, we examined markers of three human pulmonary DC subsets: myeloid DC type 1 (mDC1), myeloid DC type 2 (mDC2), and plasmacytoid DCs (pDCs). DCs were only analyzed in BAL and blood due to concerns that they would be too small of a population to identify in sputum. To identify the DC subsets, we used blood dendritic cell antigen (BDCA) markers, which we and others have previously shown accurately identifies these cell types in lung parenchyma [34,35]. First, CD45+ cells were selected followed by exclusion of cells that were positive for either CD3 or CD19+ cells, and of cells with a high forward scatter or high side-scatter. Next, mDC1 cells were identified as being double-positive for HLA-DR and BDCA-1 (CD1c), whereas mDC2 cells were identified as being double positive for HLA-DR and BDCA-3 (CD141). To identify pDC, we selected cells that were CD123+ and BDCA-2 (CD303) +.

**Figure 5:**
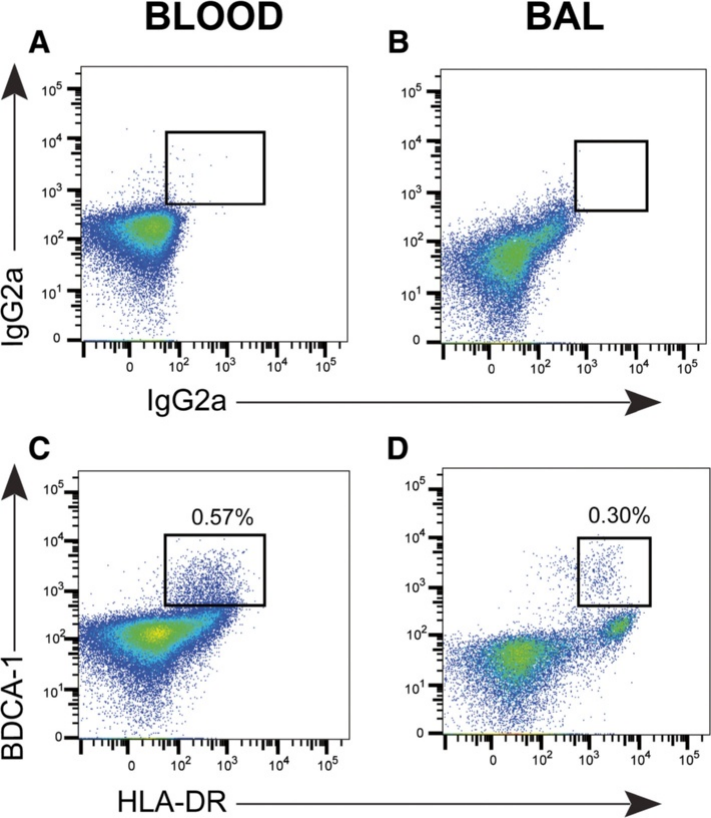
**BDCA-1 and HLA-DR identify mDC1 cells in blood and BAL.** To identify dendritic cells in samples of peripheral blood **(A, C)** and BAL **(B, D)**, we first gated on CD45+ cells, then excluded CD3+, CD19+, and cells with a high forward and side-scatter, and finally using isotype controls **(A,B)**, chose HLA-DR and BDCA-1 double-positive cells **(C,D)**. Numbers represent the percent of mDC1 cells among all CD45+ cells.

Using this method we were able to identify mDC1 subsets in blood and BAL (Figure 5). Similar results were found for mDC2 and pDCs (data not shown). CD11c, CD11b, and CD103, which are also routinely used to identify DCs, were also included in the panel for DCs. Future analyses could utilize these markers to perform more in-depth analysis of DC subsets and to compare alternative methods of DC identification.

### Identification of neutrophil populations in blood, sputum and BAL

**Figure 6:**
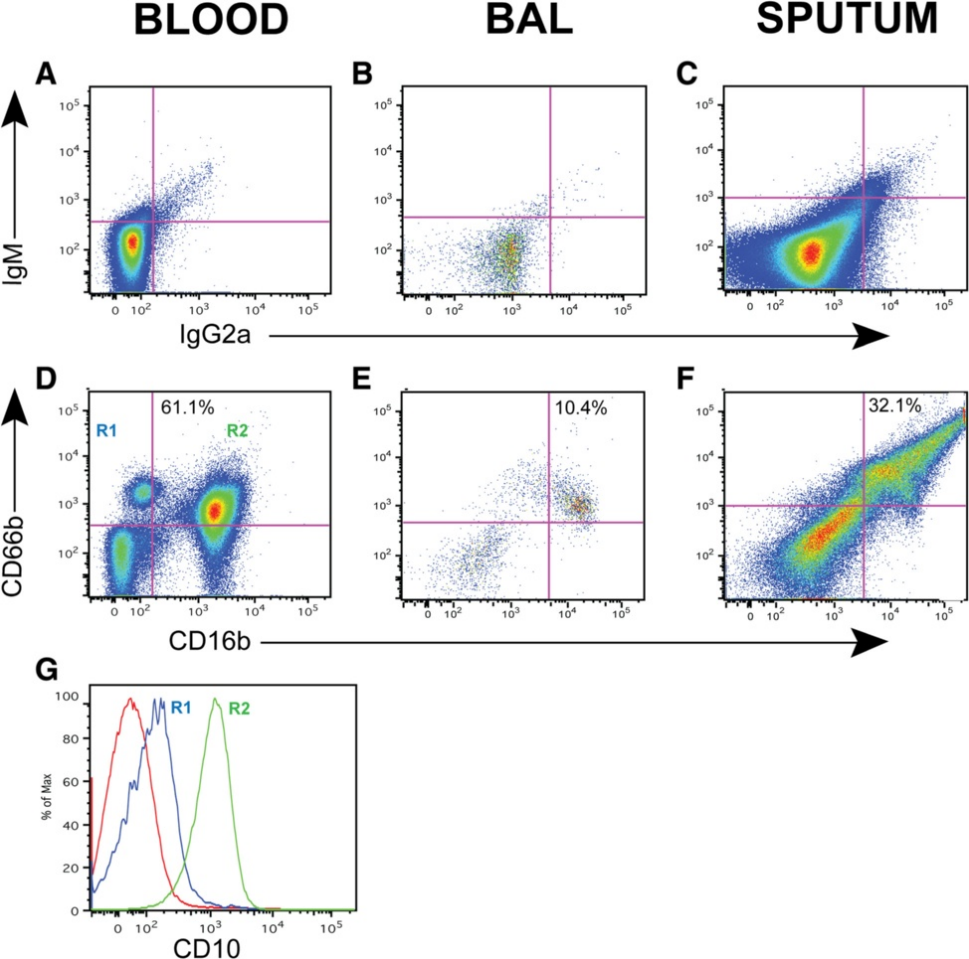
**Representative staining of neutrophils in blood, BAL, and sputum.** To identify neutrophils in samples of peripheral blood **(A, D)**, BAL **(B, E)** and sputum **(C, F)**, we gated on CD45+, high side-scatter cells that were positive for both CD16b (horizontal axis) and CD66b (vertical axis), relative to isotype controls **(A-C)**. Numbers in the quadrants **(D-F)** represent the percent of the major neutrophil population among all CD45+ cells. In the blood analysis **(panel D)**, a CD16b- (low) CD66b + population was also identified (R1). **G)** Histograms depicting expression of CD10 in the R1 (blue line) and R2 (green line) populations, relative to isotype control (red line).

The concept that neutrophils contribute centrally to emphysema stems primarily from genetic deficiency of alpha-1 antiprotease, which inhibits neutrophil elastase, although it is now recognized that this acute phase reactant has important actions independent of elastase inhibition [36]. Hence, clarifying the exact role of neutrophils in COPD phenotypes is an important goal. We identified neutrophils by gating on CD45+, high side scatter cells and then, based on appropriate isotype control (Figures 6A-6C), selected cells that were positive for two neutrophil-specific surface markers [37], CD16b and CD66b (Figure 6D-6 F). From the representative staining we saw that neutrophils were most abundant in blood and sputum samples, as expected, and were very infrequent in BAL.

In peripheral blood samples, we also observed a CD66b + CD16b- population (R1 in Figure 6D), which was not present in the BAL or sputum samples. In designing this study, we had hypothesized that in COPD subjects we might see a population of circulating neutrophils recently released from the bone marrow, identifiable as CD16b- (low) and CD10- cells [38]. We gated on both CD16b low and high populations and analyzed expression of CD10 (Figure 6B). The CD66b + CD16b- cells had reduced expression of CD10, especially in comparison to the CD66b + CD16b + cells, suggesting that we are able to identify a population of circulating immature neutrophils.

### Identification of eosinophil populations in blood, sputum and BAL

**Figure 7:**
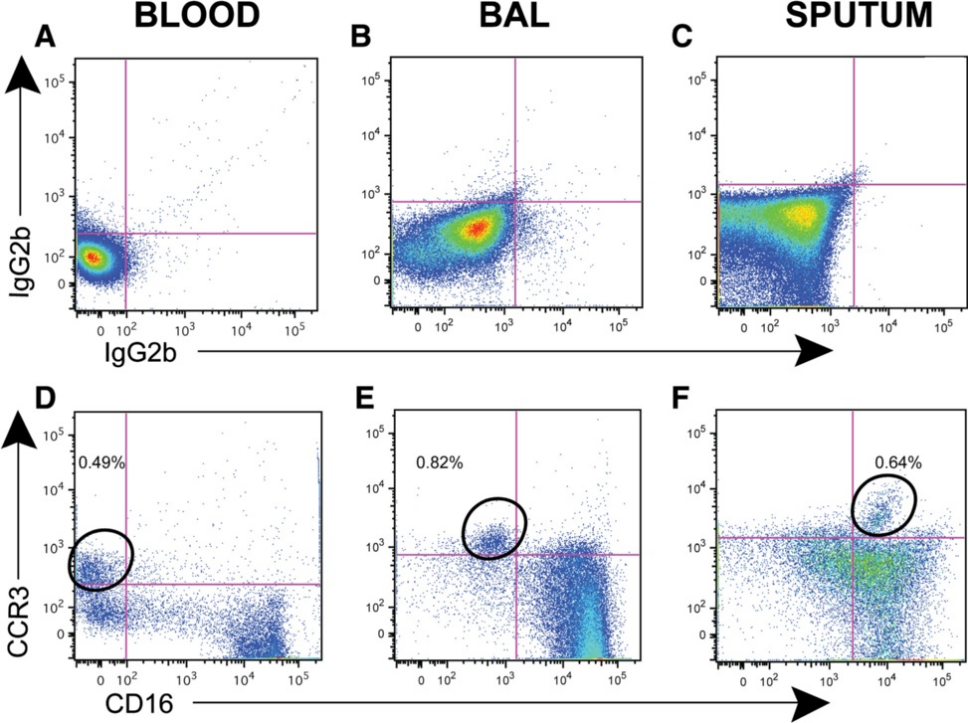
**Eosinophils in blood, BAL, and sputum express CCR3 but have variable expression of CD16.** To identify eosinophils in samples of peripheral blood **(A, D)**, BAL **(B, E)** and sputum **(C, F)**, we gated on cells that were CD45+ with a high side scatter that were also CCR3+ (vertical axis), relative to isotype controls **(A-C)**. In blood **(panel D)** and BAL samples **(panel E)**, eosinophils were also CD16- (horizontal axis). In sputum samples **(panel F)**, eosinophils were positive for CD16. Numbers represent the percent of each subset among all CD45+ cells.

Eosinophilic lung inflammation appears to identify a subset of COPD patients who are more responsive to corticosteroids or leukotriene inhibition [39-41]. We identified eosinophils in blood and BAL by first gating on CD45+ high side scatter cells and then identifying cells that, compared to isotype controls (Figure 7A-7C) were CCR3+ and CD16- [42] (Figure 7D-7F). In sputum samples, we also identified a cell population that was CCR3+ but based on the isotype control, they appeared to be positive for CD16. Eosinophils do contain intracellular CD16 and it has been shown that blood eosinophils express surface CD16 when stimulated in vitro with platelet-activating factor or IFN-γ [43]. Hence, these preliminary data are compatible with activation of eosinophils in the airways of some subjects with COPD. In future studies once we have data on all subjects, we will look for correlations between eosinophil CD16 expression and clinical characteristics.

## Discussion

This interim analysis, describing the design and early data collection of a clinical trial involving six clinical centers throughout the United States, demonstrates the feasibility of our approach of “just-in-time” provision of antibodies, distributed staining and fixation, and centralized analysis to perform 12-color immunophenotyping of samples from three different tissue origins. We have demonstrated successful identification of multiple leukocyte cell populations, including CD4+ and CD8+ T cells, monocytes, macrophages, neutrophils, and eosinophils, in blood, BAL and sputum, thereby demonstrating the validity of our antibody panel and the fixation method. We have also shown that the vast majority of samples provide usable data, supporting the feasibility of this logistic approach. This design should be considered for immunophenotyping in comparable clinical studies in other disease states.

Our approach differs from that of several other multicenter trials. The Multicenter AIDS Cohort Study (MACS) used four flow cytometry laboratories to analyze identical peripheral blood specimens. MACS identified the importance of standardizing the following variables: (1) the model of flow cytometer; (2) the antibody reagents and fluorochromes; (3) the procedure for sample preparation; and (4) the procedure for sample analysis [7]. Our approach allows us to standardize three out of those four variables. In our study, all samples are run on the same instrument, a modified LSR II flow cytometer containing a high-throughput sampler to improve efficiency of analysis and a yellow-green laser that permits use of multiple PE-conjugated fluorochromes. Data are collected and analyzed by a small team of highly trained scientists with adherence to a standardized gating strategy and frequent evaluation of the raw data and instrument controls. We have even minimized variability in the antibody reagents and fluorochromes by preparing the antibodies at the centralized core before shipping them to the Clinical Centers. In comparison, other multicenter trials have opted to combine local sample staining with the flow cytometry instruments that are available at each participating institution, although often the instruments vary in type of instrument and number of lasers [8-10]. Della Porta et al. attempted to control for this variability by including daily instrument quality controls, including fluorescence standardization, to ensure consistent operation. However, the analysis was not centrally reviewed and was performed using multiple platforms [10]. In the study by Benevolo et al., laboratories had either a 3- or 4-color instruments, which resulted in the use of different antibody panels between centers. To minimize that variable, all data in that study were reviewed by a single person [9].

Although in our study there is a single procedure for sample preparation, given that six different sites are responsible for staining the samples, there is likely some variability being introduced at this stage. We considered the idea of cryopreserving or stabilizing samples and then shipping them to a centralized flow cytometry core for staining and analysis, but ultimately decided against this approach, as the preservation of individual surface antigens is quite variable [11,12] (and our own unpublished data from pilot experiments). For the most part, our preliminary analyses have not demonstrated systematic differences between clinical centers for staining efficiency.

Another variable that we are unable to control entirely using this approach is the amount of time that the samples remain in PFA before data collection on the flow cytometer. Although we stipulate that sites send samples back as soon as possible, samples that are collected on a Thursday or Friday are not shipped until the next Monday. Excluding those days from sample collection was not compatible with the overall logistics of the Bronchoscopy sub-study. An essential feature of our study design was sample fixation using 2% paraformaldehyde post-staining to permit delayed analysis. However, paraformaldehyde itself affects flow cytometry readings. Staining with PFA has been shown to result in a slight decrease in fluorescent intensity [44] and a decrease in forward scatter and side scatter [45]. Our analysis of changes in fluorescence intensity for 68 leukocyte surface receptors for up to seven days generally agrees with a previous analysis showing that staining generally remained consistent and highly reproducible at days 1, 3 and 5 post-staining [44]. However, we found better preservation of staining intensity at day 7 than in that previous study, although two of the four receptors they studied were also stable at 7 days, suggesting that these changes are receptor-specific. Furthermore, we did not observe a change in forward scatter and side scatter. Of the molecules that were affected by storage in PFA in our study, only CD103 had decreased fluorescent intensity. The other markers that were affected, TLR4, gamma-delta T cell receptor, CCR7, BDCA-1, CX3CR1 and TLR2 actually had a slight increase in fluorescent intensity. In more detailed analyses, we will need to take this change in fluorescence into account for samples that were unable to be analyzed within 2 days.

An additional limitation of this study is the lack of a live/dead exclusion gate. FSC and SSC gating was used to eliminate cell debris, but cannot guarantee that all dead cells were excluded. A final limitation is that the protocol of the current SPIROMICS Immunophenotyping project does not include staining of intracellular antigens, isolation of specific cell types or in vitro stimulation to induce production of gene products such as cytokines or inflammatory mediators. We considered those undertakings premature until we had both demonstrated the ability of this multi-center experimental design to produce usable surface staining data, and had advanced the characterization of specific cell types in our population. However, given the technical success illustrated by these results, we believe that such extension are entirely feasible and should be an important goal of future studies in this cohort. For example, it would be of particular interest to extend previous observations supporting a role for granzyme B in the pathogenesis of emphysema [46,47] by defining which cell types harvested by BAL from area of radiographically-confirmed emphysema express that cytotoxic molecule.

Part of our future analysis strategy will be to correlate the relative proportions of a given cell type in BAL versus sputum versus blood in the same subject. Other analyses will correlate cell-surface markers on a given cell type with smoking history, spirometry, airway measurements, and other parameters. As part of the parent SPIROMICS project, individuals will also be characterized via physiological, imaging, biochemical, and genetic parameters. These data will also be available for correlating with immunophenotyping analyses.

## Conclusion

In conclusion, we have demonstrated the feasibility of providing state-of-the-art immunophenotyping of sputum, peripheral blood and BAL in a multicenter observational study of COPD biomarkers. Key features of our approach that minimize potential sources of experimental variation include “just-in-time” provision of reagents from a centralized Immunophenotyping Core, local immunostaining and fixation, and return of samples for analysis on a single flow cytometer. The choice of leukocyte cell types and their receptors was based on a series of pre-specified hypotheses plus research interests of the coauthors. This interim report demonstrates the success of our approach, which may be of value in the design of multicenter trials in other disease states. Enrollment to this sub-study is anticipated to be completed in the summer of 2015, so results should be analyzed and published over the next two years.

### Consent

Written informed consent was obtained from each participant for the publication of this report and any accompanying images.

## Abbreviations

BAL: Bronchoalveolar lavage
COPD: Chronic obstructive pulmonary disease
MACS: Multicenter AIDS Cohort Study
PFA: Paraformaldehyde
SPIROMICS: Subpopulations and Intermediate Outcomes in COPD Study

## References

[1] Minino AM, Xu J, Kochanek KD (2010). Deaths: preliminary data for 2008. Natl Vital Stat Rep.

[2] Murray CJ, Lopez AD (1997). Alternative projections of mortality and disability by cause 1990–2020: global burden of disease study. Lancet.

[3] Hogg JC, Chu F, Utokaparch S, Woods R, Elliott WM, Buzatu L, Cherniack RM, Rogers RM, Sciurba FC, Coxson HO, Pare PD (2004). The nature of small-airway obstruction in chronic obstructive pulmonary disease. N Engl J Med.

[4] Barnes PJ, Celli BR (2009). Systemic manifestations and comorbidities of COPD. Eur Respir J.

[5] Han MK, Agusti A, Calverley PM, Celli BR, Criner G, Curtis JL, Fabbri LM, Goldin JG, Jones PW, Macnee W (2010). Chronic obstructive pulmonary disease phenotypes: the future of COPD. Am J Respir Crit Care Med.

[6] Couper D, Lavange LM, Han M, Barr RG, Bleecker E, Hoffman EA, Kanner R, Kleerup E, Martinez FJ, Woodruff PG, Rennard S (2013). Design of the subpopulations and intermediate outcomes in COPD study (SPIROMICS). Thorax.

[7] Giorgi JV, Cheng HL, Margolick JB, Bauer KD, Ferbas J, Waxdal M, Schmid I, Hultin LE, Jackson AL, Park L, The Multicenter AIDS Cohort Study Group (1990). Quality control in the flow cytometric measurement of T-lymphocyte subsets: the multicenter AIDS cohort study experience. Clin Immunol Immunopathol.

[8] Bradstock K, Matthews J, Benson E, Page F, Bishop J (1994). Prognostic value of immunophenotyping in acute myeloid leukemia. Blood.

[9] Benevolo G, Stacchini A, Spina M, Ferreri AJ, Arras M, Bellio L, Botto B, Bulian P, Cantonetti M, Depaoli L (2012). Final results of a multicenter trial addressing role of CSF flow cytometric analysis in NHL patients at high risk for CNS dissemination. Blood.

[10] Della Porta MG, Picone C, Pascutto C, Malcovati L, Tamura H, Handa H, Czader M, Freeman S, Vyas P, Porwit A (2012). Multicenter validation of a reproducible flow cytometric score for the diagnosis of low-grade myelodysplastic syndromes: results of a European LeukemiaNET study. Haematologica.

[11] Ng AA, Lee BT, Teo TS, Poidinger M, Connolly JE (2012). Optimal cellular preservation for high dimensional flow cytometric analysis of multicentre trials. J Immunol Methods.

[12] Davis C, Wu X, Li W, Fan H, Reddy M (2011). Stability of immunophenotypic markers in fixed peripheral blood for extended analysis using flow cytometry. J Immunol Methods.

[13] Alexis N, Soukup J, Ghio A, Becker S (2000). Sputum phagocytes from healthy individuals are functional and activated: a flow cytometric comparison with cells in bronchoalveolar lavage and peripheral blood. Clin Immunol.

[14] Majori M, Corradi M, Caminati A, Cacciani G, Bertacco S, Pesci A (1999). Predominant T_h1_ cytokine pattern in peripheral blood from subjects with chronic obstructive pulmonary disease. J Allergy Clin Immunol.

[15] Grumelli S, Corry DB, Song LZ, Song L, Green L, Huh J, Hacken J, Espada R, Bag R, Lewis DE, Kheradmand F (2004). An immune basis for lung parenchymal destruction in chronic obstructive pulmonary disease and emphysema. PLoS Med.

[16] Sullivan AK, Simonian PL, Falta MT, Mitchell JD, Cosgrove GP, Brown KK, Kotzin BL, Voelkel NF, Fontenot AP (2005). Oligoclonal CD4+ T cells in the lungs of patients with severe emphysema. Am J Respir Crit Care Med.

[17] Freeman CM, Curtis JL, Chensue SW (2007). CC chemokine receptor 5 and CXC chemokine receptor 6 expression by lung CD8+ cells correlates with chronic obstructive pulmonary disease severity. Am J Pathol.

[18] Lee SH, Goswami S, Grudo A, Song LZ, Bandi V, Goodnight-White S, Green L, Hacken-Bitar J, Huh J, Bakaeen F (2007). Antielastin autoimmunity in tobacco smoking-induced emphysema. Nat Med.

[19] Freeman CM, Martinez FJ, Han MK, Washko GR, McCubbrey AL, Chensue SW, Arenberg DA, Meldrum CA, McCloskey L, Curtis JL (2013). Lung CD8+ T cells in COPD have increased expression of bacterial TLRs. Respir Res.

[20] Lay JC, Peden DB, Alexis NE (2011). Flow cytometry of sputum: assessing inflammation and immune response elements in the bronchial airways. Inhal Toxicol.

[21] Leckie MJ, Jenkins GR, Khan J, Smith SJ, Walker C, Barnes PJ, Hansel TT (2003). Sputum T lymphocytes in asthma, COPD and healthy subjects have the phenotype of activated intraepithelial T cells (CD69+ CD103+). Thorax.

[22] Heron M, Grutters JC, ten Dam-Molenkamp KM, Hijdra D, van Heugten-Roeling A, Claessen AM, Ruven HJ, van den Bosch JM, van Velzen-Blad H (2012). Bronchoalveolar lavage cell pattern from healthy human lung. Clin Exp Immunol.

[23] Hiemstra PS (2013). Altered macrophage function in chronic obstructive pulmonary disease. Ann Am Thorac Soc.

[24] Woodruff PG, Koth LL, Yang YH, Rodriguez MW, Favoreto S, Dolganov GM, Paquet AC, Erle DJ (2005). A distinctive alveolar macrophage activation state induced by cigarette smoking. Am J Respir Crit Care Med.

[25] Shaykhiev R, Krause A, Salit J, Strulovici-Barel Y, Harvey BG, O'Connor TP, Crystal RG (2009). Smoking-dependent reprogramming of alveolar macrophage polarization: implication for pathogenesis of chronic obstructive pulmonary disease. J Immunol.

[26] Gautier EL, Shay T, Miller J, Greter M, Jakubzick C, Ivanov S, Helft J, Chow A, Elpek KG, Gordonov S (2012). Gene-expression profiles and transcriptional regulatory pathways that underlie the identity and diversity of mouse tissue macrophages. Nat Immunol.

[27] Guth AM, Janssen WJ, Bosio CM, Crouch EC, Henson PM, Dow SW (2009). Lung environment determines unique phenotype of alveolar macrophages. Am J Physiol Lung Cell Mol Physiol.

[28] Ziegler-Heitbrock L (2007). The CD14+ CD16+ blood monocytes: their role in infection and inflammation. J Leukoc Biol.

[29] Chow A, Brown BD, Merad M (2011). Studying the mononuclear phagocyte system in the molecular age. Nat Rev Immunol.

[30] Moniuszko M, Bodzenta-Lukaszyk A, Kowal K, Lenczewska D, Dabrowska M (2009). Enhanced frequencies of CD14++CD16+, but not CD14 + CD16+, peripheral blood monocytes in severe asthmatic patients. Clin Immunol.

[31] Ziegler-Heitbrock L, Hofer TP (2013). Toward a refined definition of monocyte subsets. Front Immunol.

[32] Hijdra D, Vorselaars AD, Grutters JC, Claessen AM, Rijkers GT (2013). Phenotypic characterization of human intermediate monocytes. Front Immunol.

[33] Brittan M, Barr L, Conway Morris A, Duffin R, Rossi F, Johnston S, Monro G, Anderson N, Rossi AG, McAuley DF (2012). A novel subpopulation of monocyte-like cells in the human lung after lipopolysaccharide inhalation. Eur Respir J.

[34] Demedts IK, Brusselle GG, Vermaelen KY, Pauwels RA (2005). Identification and characterization of human pulmonary dendritic cells. Am J Respir Cell Mol Biol.

[35] Freeman CM, Martinez FJ, Han MK, Ames TM, Chensue SW, Todt JC, Arenberg DA, Meldrum CA, Getty C, McCloskey L, Curtis JL (2009). Lung dendritic cell expression of maturation molecules increases with worsening chronic obstructive pulmonary disease. Am J Respir Crit Care Med.

[36] Jonigk D, Al-Omari M, Maegel L, Muller M, Izykowski N, Hong J, Hong K, Kim SH, Dorsch M, Mahadeva R (2013). Anti-inflammatory and immunomodulatory properties of alpha1-antitrypsin without inhibition of elastase. Proc Natl Acad Sci U S A.

[37] Wright AK, Rao S, Range S, Eder C, Hofer TP, Frankenberger M, Kobzik L, Brightling C, Grigg J, Ziegler-Heitbrock L (2009). Pivotal advance: expansion of small sputum macrophages in CF: failure to express MARCO and mannose receptors. J Leukoc Biol.

[38] Orr Y, Taylor JM, Bannon PG, Geczy C, Kritharides L (2005). Circulating CD10-/CD16low neutrophils provide a quantitative index of active bone marrow neutrophil release. Br J Haematol.

[39] Brightling CE, McKenna S, Hargadon B, Birring S, Green R, Siva R, Berry M, Parker D, Monteiro W, Pavord ID, Bradding P (2005). Sputum eosinophilia and the short term response to inhaled mometasone in chronic obstructive pulmonary disease. Thorax.

[40] Brightling CE, Monteiro W, Ward R, Parker D, Morgan MD, Wardlaw AJ, Pavord ID (2000). Sputum eosinophilia and short-term response to prednisolone in chronic obstructive pulmonary disease: a randomised controlled trial. Lancet.

[41] Siva R, Green RH, Brightling CE, Shelley M, Hargadon B, McKenna S, Monteiro W, Berry M, Parker D, Wardlaw AJ, Pavord ID (2007). Eosinophilic airway inflammation and exacerbations of COPD: a randomised controlled trial. Eur Respir J.

[42] Stein ML, Villanueva JM, Buckmeier BK, Yamada Y, Filipovich AH, Assa'ad AH, Rothenberg ME (2008). Anti-IL-5 (mepolizumab) therapy reduces eosinophil activation ex vivo and increases IL-5 and IL-5 receptor levels. J Allergy Clin Immunol.

[43] Davoine F, Labonte I, Ferland C, Mazer B, Chakir J, Laviolette M (2004). Role and modulation of CD16 expression on eosinophils by cytokines and immune complexes. Int Arch Allergy Immunol.

[44] Atar OD, Eisert C, Pokov I, Serebruany VL (2010). Stability validation of paraformaldehyde-fixed samples for the assessment of the platelet PECAM-1, P-selectin, and PAR-1 thrombin receptor by flow cytometry. J Thromb Thrombolysis.

[45] Stewart JC, Villasmil ML, Frampton MW (2007). Changes in fluorescence intensity of selected leukocyte surface markers following fixation. Cytometry A.

[46] Hodge S, Hodge G, Nairn J, Holmes M, Reynolds PN (2006). Increased airway granzyme B and perforin in current and ex-smoking COPD subjects. COPD.

[47] Kim WD, Chi HS, Choe KH, Oh YM, Lee SD, Kim KR, Yoo KH, Ngan DA, Elliott WM, Granville DJ (2013). A possible role for CD8+ and non-CD8+ cell granzyme B in early small airway wall remodelling in centrilobular emphysema. Respirology.

